# Genome-Wide Identification and Expression Analysis of the Protease Inhibitor Gene Families in Tomato

**DOI:** 10.3390/genes11010001

**Published:** 2019-12-18

**Authors:** Yuxuan Fan, Wei Yang, Qingxia Yan, Chunrui Chen, Jinhua Li

**Affiliations:** 1 Key Laboratory of Horticulture Science for Southern Mountainous Regions, Ministry of Educatio, College of Horticulture and Landscape Architecture, Southwest University, No.2 Tiansheng Road, Beibei, Chongqing 400715, China; fanyuuxan123@email.swu.edu.cn (Y.F.); shucaitomato@gmail.com (W.Y.); yanqx1998@hotmail.com (Q.Y.); eline1020@email.swu.edu.cn (C.C.); 2 State Cultivation Base of Crop Stress Biology for Southern Mountainous land of Southwest University, Academy of Agricultural Sciences, Southwest University, Beibei, Chongqing 400715, China

**Keywords:** tomato, protease inhibitor, gene family, abiotic and biotic stress, expression profiles

## Abstract

The protease inhibitors (PIs) in plants are involved primarily in defense against pathogens and pests and in response to abiotic stresses. However, information about the PI gene families in tomato (*Solanum lycopersicum*), one of the most important model plant for crop species, is limited. In this study, in silico analysis identified 55 *PI* genes and their conserved domains, phylogenetic relationships, and chromosome locations were characterized. According to genetic structure and evolutionary relationships, the PI gene families were divided into seven families. Genome-wide microarray transcription analysis indicated that the expression of *SlPI* genes can be induced by abiotic (heat, drought, and salt) and biotic (*Botrytis cinerea* and tomato spotted wilt virus (TSWV)) stresses. In addition, expression analysis using RNA-seq in various tissues and developmental stages revealed that some *SlPI* genes were highly or preferentially expressed, showing tissue- and developmental stage-specific expression profiles. The expressions of four representative *SlPI* genes in response to abscisic acid (ABA), salicylic acid (SA), ethylene (Eth), gibberellic acid (GA). and methyl viologen (MV) were determined. Our findings indicated that PI genes may mediate the response of tomato plants to environmental stresses to balance hormone signals. The data obtained here will improve the understanding of the potential function of PI gene and lay a foundation for tomato breeding and transgenic resistance to stresses.

## 1. Introduction

Protease is one of the most important enzymes involved in various physiological and biochemical processes, such as protein catabolism, signal transduction, digestion, and protein–protein interaction, in all organisms [1]. Proteases are critical for the maintenance and survival of their host organisms, but their high activity may be damaging. Therefore, it is crucial to regulate protease activity for maintaining the cellular homeostasis. Among several methods controlling excessive protease activity, the use of protease inhibitors (PIs) is a promising approach. PIs effectively modulate protease activity by direct interaction and clearing the signal receptor of the proteases [2,3].

PIs are widely distributed in the plant kingdom [4]. Plant PIs, usually small proteins, are present in storage tissues, such as tubers and seeds, but they have also been found in the aerial parts of plants [5]. Plant PIs are localized in plant tissues, including leaves, stems, and developing fruits and seedlings. By analysis of plant PIs development- and tissue-specific expression, their physiological functions have been predicted [6]. 

On the basis of the specificity of the target protease, PIs are divided into aspartic acid PI, serine PI (serpins), cysteine PI (cystatins), and metallocarboxy PI [7]. Based on structural and biochemical properties, PIs are also classified as serpins and Bowman-Birk serine, cysteine, and potato type I and type II PIs, cereal trypsin/*α*-amylase, mustard trypsin, metallocarboxypeptidase, soybean trypsin (Kunitz), and squash inhibitors [8]. The 48 identified PIs are grouped into 26 related superfamilies (or clans) on the basis of their structure through their amino acid sequence similarities [9]. According to the MEROPS database, the inhibitors have 82 family members [10].

Plant PIs play a vital role in defenses against pests and pathogens, especially against herbivorous pests [11,12,13,14]. Green and Ryan first observed that plant PIs prevented insect predation and accumulated rapidly in potatoes (*Solanum tuberosum*) and tomatoes (*Solanum lycopersicum*) when their leaves were attacked by Colorado potato beetles [15]. Since then, the role of PIs in plants has been extensively studied through biochemical analysis, transgenic plant research, and insect feeding experiments [7,16,17,18,19,20,21,22,23]. For instance, transgenic rice and tobacco introduced with the potato *PI II* gene (pin2) have increased resistance to the main rice pests, *Sesamia inferens* and *Lepidopteran Manduca sexta* [21,24]. In transgenic tomatoes, the overexpression of cysteine PIs from rice has conferred tolerance to cyst nematode [25]. Generally, PIs can inhibit the protein digestive enzymes in the gut of insects, resulting in amino acid deficiency and subsequent delayed development, death, and/or reduced fecundity [26]. Despite the detrimental effects of PIs on insect pests, the long-term coevolution of pests and plants has promoted resistance to many PI genes, making countless plant defensive protease collections almost worthless for pest management. Sometimes, even insects need to consume transgenic plant materials to compensate for nutritional stress [19,27,28].

PIs derived from plants are multifunctional proteins involved in plant protection against pathogens and herbivores and in plant tolerance modulation to diverse abiotic stresses. The Kunitz family PIs are upregulated in NaCl-treated *Arabidopsis* and radish under drought stress [29,30]. The PI genes in chestnut, rice, and barley had a positive effect on drought resistance [31,32,33]. After high salt treatment, the transcription level of the PI-II gene (*CaPI-2*) was upregulated in red pepper [30]. The trypsin PI gene (*NtPI*) in tobacco increased tolerance to various stresses, including salinity, drought, and pH [34]. Under various abiotic stresses, such as salt, osmosis, oxidation, and cold stresses, the mRNA level of ApCystatin was significantly upregulated, indicating that ApCystatin is involved in plant stress tolerance [35]. 

The production of PI is highly regulated by a signal transduction pathway [36]. Growth regulators, such as jasmonic acid (JA), abscisic acid (ABA), ethylene (Eth), and salicylic acid (SA), modulate defense responses by attenuating or stimulating signal transduction [37]. SA plays an important role in the defense response of plants to abiotic and biotic stresses by controlling the cysteine PI gene (*SAG1*2) [38]. The expression levels of ABA and PI simultaneously increase in response to wounding, electrical signals, heat treatment, or systemic applications. These growth regulators can participate in plant resistance to biotic and abiotic stresses by modulating the signaling pathway of PI expression. 

PIs are involved in biotic and abiotic stresses, but their detailed function remains to be determined. As far as we know, the whole genome of the PI protein has not been investigated in plants. In order to enrich the information of PI gene families in tomato genome level, the members of the PI gene families in tomato were identified using bioinformatics methods, and their gene family classification, exon-intron structure, phylogenetic comparison of chromosome distribution, and *cis*-acting elements were analyzed. Moreover, the expression patterns under various hormones, stress treatments, and tissues/stages were investigated. The study of PI in different families in tomato and their possible functions were also discussed. These findings offer valuable information that provides a solid basis for further characterization of the function of PI in tomato and detailed molecular mechanisms.

## 2. Materials and Methods

### 2.1. Plant Materials and Real Time PCR

Tomato (*S. lycopersicum* cv. M82) plants were grown in a greenhouse with a 16 h light/8 h dark cycle. Five-leaf stage tomato plants were subjected to plant hormone and oxidative stress treatments (sprayed with 100 μM ABA, 100 μM GA, 100 μM SA, 1 mM Eth, and 100 μM MV until runoff) simultaneously for gene expression profiling. Untreated plants were used as controls to avoid the effects of biological clock on differential gene expression. After 0, 1, 4, 6, 12, and 24 h of treatment, the materials of leaves were immediately frozen in liquid nitrogen and stored at −80 °C. The RNA isolation and real-time PCR was performed as described previously [39] with gene-specific primers (Supplementary Table S1). 

### 2.2. Identification of Tomato PI Genes 

The predicted PI genes were identified as follows. First, homologs were searched using “protease inhibitor” as a keyword in the SGN (Sol Genomics Network) tomato database. Afterward, the PI genes were verified and found via the BLASTP search against the SGN and National Center for Biotechnology Information databases. After manually removing the redundant sequences, all these predicted genes were examined for the different PI domains in SMART (version 7, EMBL, Heidelberg, Germany), Pfam (version 32, EMBL-EBI, Cambridgeshire, UK), and InterProScan (version 71, EMBL-EBI, Cambridgeshire, UK), and those without the different PI domains were excluded.

### 2.3. Phylogenetic Analysis

The PI families’ protein sequence of tomato (downloaded from SGN) was analyzed using the ClustalX software (version 2.1, UCD, Dublin, Ireland). Based on the results of aa sequence alignment, the neighbor-joining algorithm in MEGA (version 6, Pennsylvania State University, State College, PA, USA) [40] was used to draw the phylogenetic tree. The mode and gap was set to "Poisson correction" and "Pairwise Deletion", respectively, and the verification parameter was Bootstrap = 1000.

### 2.4. Chromosomal Location, Gene Structure, and Sequence Alignment

The PI genes were mapped to tomato chromosomes by identifying their chromosomal positions according to SGN database. The exon and intron structures of *SlPIs* were generated using the Gene Structure Display Server 2.0 [41] by aligning the CDS sequences with the corresponding genomic DNA sequences from SGN database. Domains were identified using the Pfam and the SMART programs.

### 2.5. Cis-Element Prediction for PI Gene Promoter

The PI sequences were used as BLASTN (version 2.40, NCBI, Bethesda, MD, USA) search queries against the tomato whole genome scaffolds at the SGN website. The promoter sequences (3.0 kb upstream of 5′UTR) of all identified PI genes were submitted to the PlantCARE (Plant *Cis*-Acting Regulatory Element) database for *cis*-element prediction. 

### 2.6. Tissue Expression Analysis of SlPIs of Tomato

The IDs of *SlPI* genes were searched in the online NexGenEx-Tom database [42] to investigate their expression patterns. Data showing the *SlPI* expression in 10 different tissues/stages, including leaf, root, flower, flower bud, 1, 2, and 3 cm fruit, mature green fruit, breaker fruit, and fruit at 10 days were obtained from two biological replicates. The gene expression level was defined on the basis of the iTAG mRNA loci and normalized using reads per kilobase per million (RPKM) for each tissue/stage, and the log2 transformation was selected from the platform [42]. 

### 2.7. Expression Pattern of SlPI Genes Under Different Stress Conditions

Microarray analysis was performed to gain insight into the expression profiles of the *SlPI* gene families under different environmental stresses. Whole genome microarray data for various environmental stresses, such as drought, salt, high temperature, *B. cinerea*, and tomato spotted wilt virus (TSWV)) were obtained from the TFGD (Tomato Functional Genomics Database) database. The array platforms for microarray data included the TOM2 oligo and the Affymetrix genome arrays. For the TOM2 oligo array, the probe sets of the *SlPI* genes were identified through BlastN analysis in the database “TOM2 oligo sequences”. For the Affymetrix genome array, the probe sets of the *SlPI* genes were identified through BlastN analysis in the database “Affy target sequences”. The average value was considered for *SlPI* genes with more than one probe set. The expression values of *SlPI* genes that were upregulated or downregulated by more than two-fold with *p* < 0.05 were considered as differently expressed.

## 3. Results

### 3.1. Identification of PI Genes in Tomato

In this study, 55 PI genes in the tomato genome cDNA release 2.40 were identified (Table 1). These genes were named as *SlPI01* to *SlPI55* on the basis of their distributions and relative linear orders among their respective chromosomes. To further understand PI proteins, the amino acid (aa) length, molecular weight, isoelectric point, hydrophilic coefficient, and subcellular localization prediction of 55 PI proteins were analyzed (Table 1). The lengths of SlPI proteins varied from 77 aa residues (SlPI04, SlPI29, and SlPI30) to 603 aa residues (SlPI25), with an average length of 179 aa. Most of the lengths of the PI proteins were between 100 and 300 aa. Molecular weight ranged from 8 kDa (SlPI04) to 68 kDa (SlPI25), and the isoelectric point varied from 4.64 (SlPI30) to 10.11 (SlPI47). Most tomato PI proteins (72.7%) were predicted to be in the extracellular matrix, but some proteins may be located in other subcellular compartments, such as the cytoplasm (14.5%), chloroplasts (10.9%), and vacuoles (1.8%).

### 3.2. Conserved Domain and Phylogenetic Analyses of the Tomato PI Families

**Figure 1:**
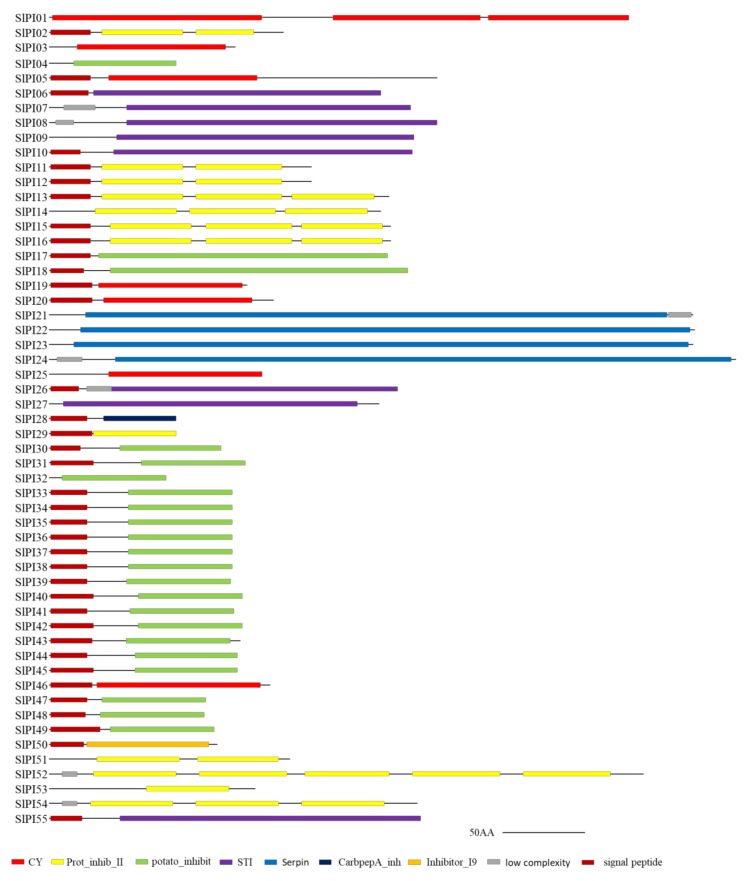
Conserved domains of tomato PI protein. Gray lines represent amino acid sequences, and each domain is indicated by a colored box. Scale bar represents 50 amino acids. The lengths of the domain in each protein are drawn to scale. Domains were identified using the SMART (http://smart.embl-heidelberg.de/) and the Pfam (http://pfam.sanger.ac.uk/) programs.

All identified tomato PI proteins contained at least one domain related to PIs (Supplementary Table S2). Seven domains related to PIs, namely, Cystatin-like domain (CY), potato peptidase inhibitor II (prot_inhib_II), soybean trypsin inhibitor (STI), serpins, potato peptidase inhibitor I (potato_inhibit), CarbpepA_inh, and Inhibitor_I9, were found in 55 SlPI proteins (Figure 1). The numbers of PI proteins containing CY, Prot_inhib_II, STI, serpin, potato_inhibit were 7, 12, 8, 4, and 22, respectively. The CarbpepA_inh only existed in SlPI28, and Inhibitor_I9 only existed in SlPI50.

**Figure 2:**
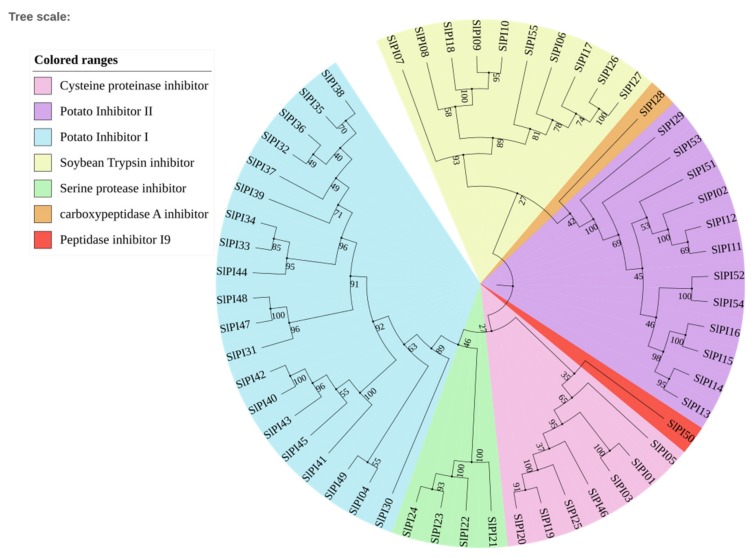
Phylogenetic analysis of the tomato PI families. Trees were based on the protein sequence alignments with the full-length PI protein sequence. Different colors of the sectors indicate the different subfamily members according to sequence similarity annotation analysis. The phylogenetic tree was constructed using the MEGA6 program by using the neighbor-joining method at 1000 bootstrap replicates. Amino acid sequence comparison of *SlPI* genes was shown in Supplementary Figure S1.

PIs with single inhibitor domains are dubbed simple inhibitors, and those with multiple inhibitor domains are called complex inhibitors. All 55 SlPI proteins are simple inhibitors. Moreover, PI proteins with the same domain were similar and concentrated. For example, all potato_inhibit domains were in the C-terminal. In summary, the diversity of domain type caused the functional diversity of PI in tomato. To explore the phylogenetic relationship and divergence of the PI families in tomato, a phylogenetic tree was constructed using the MEGA6 in accordance with the aligned 55 PI protein sequences. According to the phylogenetic analysis, the tomato PI families were divided into seven families, including Potato Inhibitor I, Potato Inhibitor II, Cysteine proteinase inhibitor, Serine protease inhibitor, Soybean trypsin inhibitor (Kunitz), carboxypeptidase A inhibitor, and Peptidase inhibitor I9 (Figure 2). Except for SlPI17 and SlPI18, the PI proteins located in the same subfamily had the same domain. The potato inhibitor I subfamily was the largest with 20 members, whereas the potato inhibitor II, cysteine PI, s serpin, and STI had 12, 7, 4, and 10 members, respectively. 

### 3.3. Chromosomal Localization, Gene Structure, and Duplication of SlPIs

**Figure 3:**
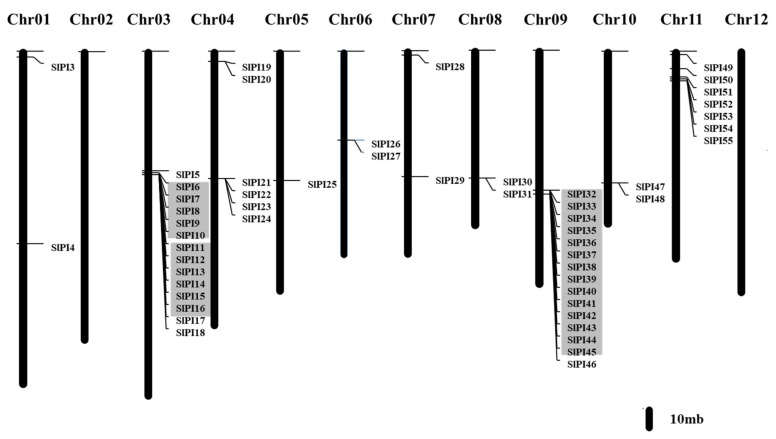
Chromosome distribution and duplication events of tomato PI genes. Chromosome localization is based on the physical location (Mb) of 12 tomato chromosomes. Chromosome numbers are displayed at the top of each bar chart. The locations of tomato PI genes in chromosomes were obtained from the Sol Genomics Network database (http://solgenomics.net). The ratio represents 10 Mb chromosomal distance. Gray squares represent tandem repeat genes.

The PI genes in tomatoes were unevenly distributed across chromosomes and mostly existed in the form of gene clusters. The tomato PI genes were mapped with the published chromosomes of tomato genome to determine their genomic distribution (Figure 3). The chromosomal localizations of two *SlPIs*, namely, *SlPI01* and *SlPI02*, were uncertain, and the other *SlPIs* were distributed in 10 chromosomes. Notably, these *SlPI* genes were unevenly distributed in chromosomes. High-density regions harboring PIs were discovered in chromosomes 3, 4, 9, and 11. Chromosome 9 contained the most PI genes. Only one PI gene (*SlPI25*) was found in chromosome 5, whereas two PI genes were found in chromosomes 1, 6, 7, 8, and 10.

**Figure 4:**
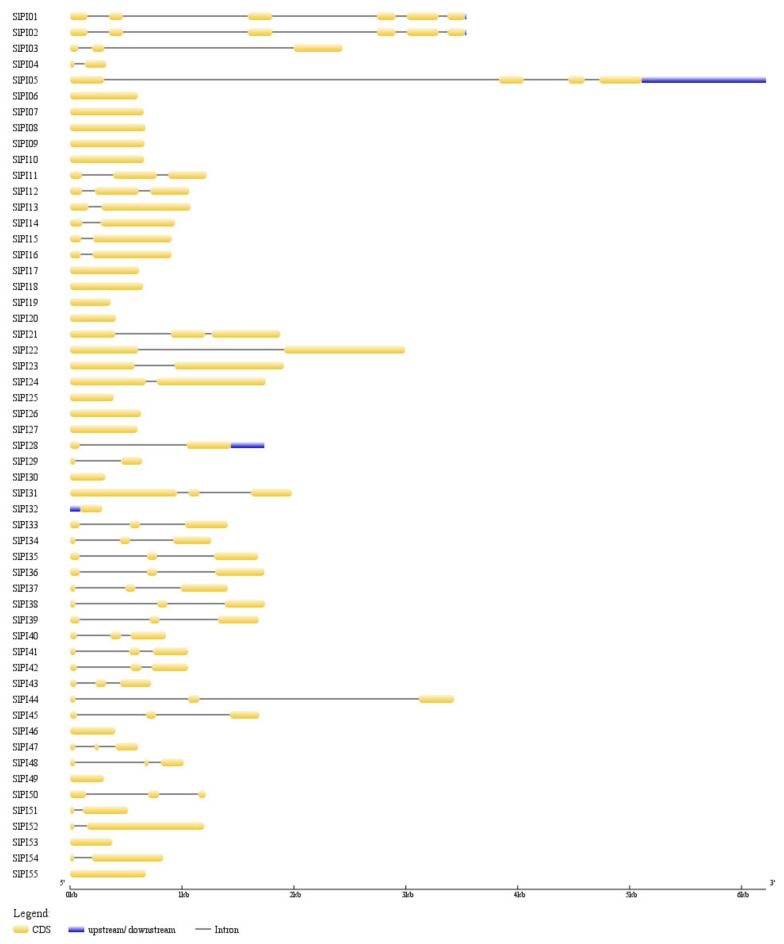
Exon-intron structures of the tomato PI families. The yellow block indicates the coding sequence, and the blue block refers to the upstream or downstream of genes. The solid line represents the intron. Scale bar indicates DNA sequence length.

As shown in Figure 4, the tomato PI gene exon-intron organizations were diverse. Most *SlPI* genes (94.54%) contained 1–3 exons. *SlPI05*, *SlPI01*, and *SlPI02* contained 4, 5, and 5 exons. Out of the 55 *SlPIs*, 21 (38.19%) had three exons, and 18 (32.73%) had no intron. Furthermore, the members of a subfamily showed structural similarities. All PI genes in the STI subfamily (*SlPI06*, *SlPI07*, *SlPI08, SlPI09*, *SlPI10*, *SlPI17*, *SlPI18*, *SlPI26*, *SlPI27*, and *SlPI55*) had no intron. 

### 3.4. Cis-Elements in the Promoters of Tomato PI Genes

**Figure 5:**
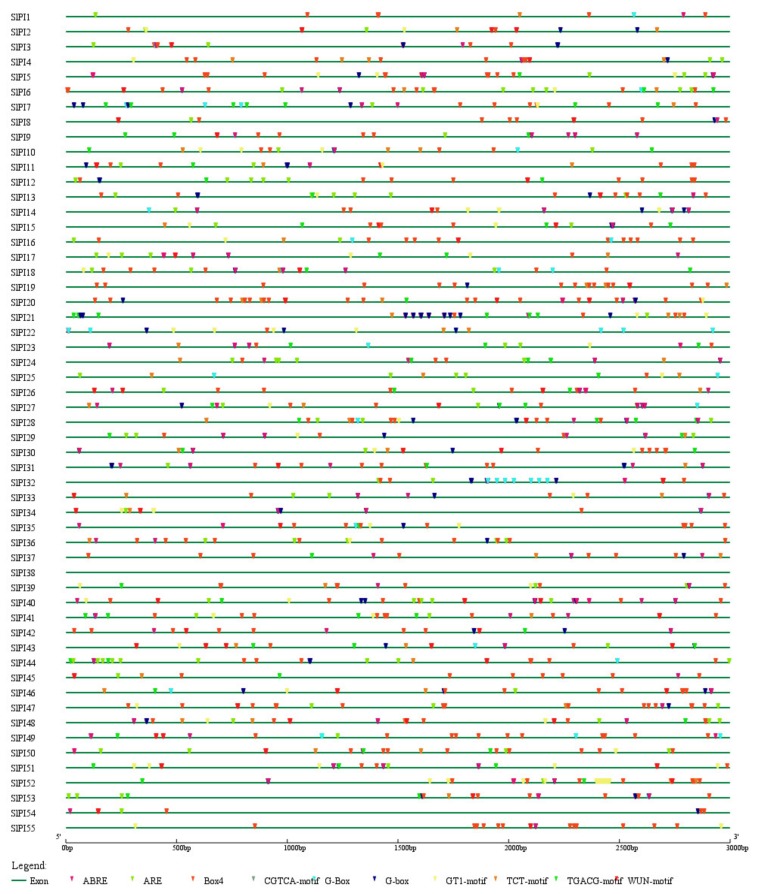
Identified cis-elements in the promoters of *SlPI* genes. The green line represents the upstream of the *SlPI* gene. Different colored wedges represent different cis-elements. The length and position of each *SlPI* gene are drawn to scale. Scale bar indicates DNA sequence length.

The gene transcription levels were regulated by the interaction of transcription factors with the cis-acting element in the upstream promoter sequences. Therefore, studying the cis-element in the promoter of PI genes in the tomato may help explore the function of *SlPI* genes. The promoter regions of 55 *SlPI* genes were analyzed using the online software Plant CARE (Supplementary Table S3). A total of 43 cis-elements were identified in more than 20 PI genes (Table 2 and Supplementary Table S4). Among the 43 cis-elements, the functions of 22 cis-elements were not annotated. By excluding the common cis-elements, such as the TATA-box and CAAT-box, the 19 remaining cis-elements can be divided into four groups. Eight cis-elements, namely, Box4, G-box, G-Box, TCT-motif, GT1-motif, GATA-motif, I-box, and chs-CMA1a, were light-responsive. Five cis-elements, including ABRE, CGTCA-motif, TGACG-motif, TCA-element, CAT-box, and five stress-related elements, including ARE, WUN-motif, TC-rich repeats, MBS, and LTR, were related to hormones. Furthermore, the CAT-box, the only element in group 4, was related to meristem expression. Notably, many cis-elements related to abiotic stress in plants were identified at the promoter of PI gene (Figure 5), and the promoter of 52 PI genes had ABRE (cis-element involved in the ABA responsiveness), indicating that PIs played an important role in abiotic stress resistance via ABA response. 

### 3.5. Expression Patterns of SlPI Genes Induced by Different Abiotic and Biotic Stresses

**Figure 6:**
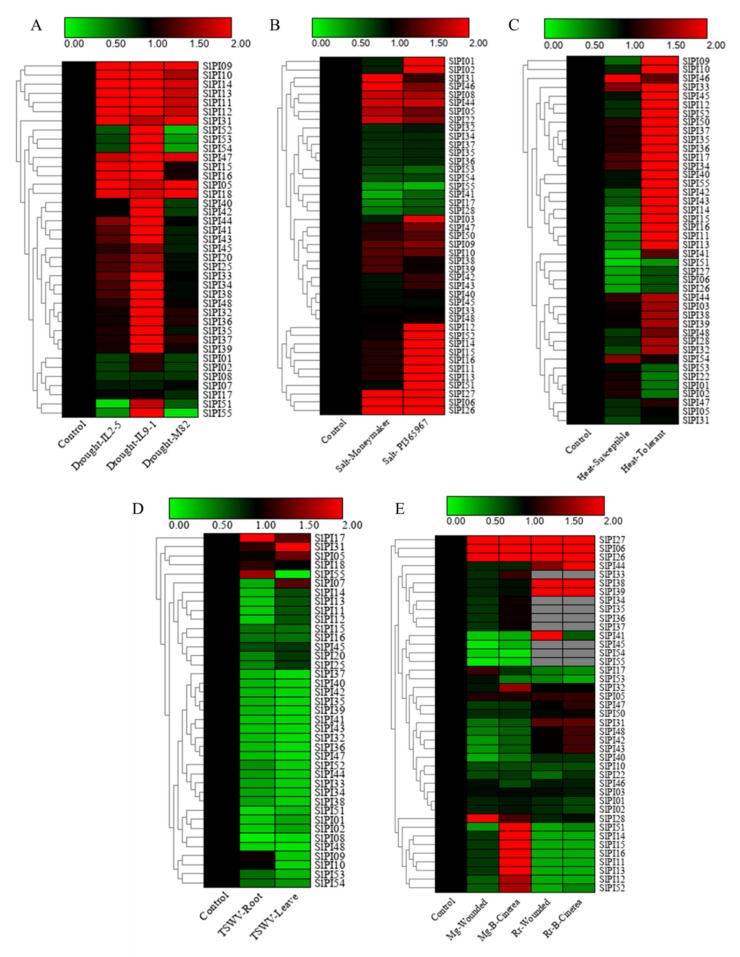
Expression profiles of *SlPI* genes under various biotic and abiotic stress conditions. Blocks with colors represent decreased (green) or increased (red) transcript levels relative to the control. (**A**) Expression profiles of *SlPIs* under drought stress condition in two drought-tolerant lines (IL2-5 and IL9-1) and a drought-sensitive cultivar (M82). (**B**) Expression profiles of *SlPI* genes under salt stress in a wild type tomato genotype “PI365967” (salt-tolerant) and cultivated tomato variety, Moneymaker (salt-sensitive). (**C**) Expression profiles of *SlPIs* in tolerant and susceptible tomatoes under heat stress condition. (**D**) Expression profiles of *SlPIs* in tomato roots and leaves infected by tomato spotted wilt virus (TSWV). (**E**) Expression profiles of *SlPIs* infected by wound and wound-inoculated with *Botrytis cinerea* in mature green (Mg) and red fruits (Rr). Gray box indicates that raw reads were not detected.

Five tomato microarray datasets from the Tomato Functional Genomics Database were obtained, which belonged to two array platforms (TOM2 oligonucleotide arrays and Affymetrix genome array), were obtained to further investigate the expression patterns of *SlPI* genes under various abiotic and biotic stresses (Figure 6). A total of 48 *SlPI* genes (87.3%) corresponding to probes were found, whereas 37 *SlPI* genes (70.1%) showed different cross-reactive probes (Supplementary Table S5).

The microarray-based expression analysis revealed that the expression of most *SlPI* genes in tomato were highly variable under various abiotic stresses (Figure 6A–C). The transcript levels of 12, 34, and 10 *SlPI* genes were upregulated, whereas those of 8, 2, and 10 *SlPI* genes were downregulated in drought-tolerant tomato lines (IL2-5 and IL9-1) and drought-sensitive varieties (M82) under drought conditions (Figure 6A). Ten *SlPI* genes (*SlPI09, SlPI10, SlPI14, SlPI13, SlPI11, SlPI12, SlPI31, SlPI47, SlPI05*, and *SlPI18*) showed an increase in transcription levels in the three tested tomato genotypes. Under salt-treated conditions, 10 *SlPI* genes (*SlPI17, SlP28, SlPI34, SlPI35, SlPI36, SlPI37, SlPI41, SlPI53, SlPI54*, and *SlPI55*) were downregulated (Figure 6B). PI365967, a highly salt-tolerant tomato genotype, showed more upregulated *SlPI* genes than the tomato cultivar, Moneymaker. The expression of 13 genes (*SlPI13, SlPI11, SlPI16, SlPI15, SlPI14, SlPI43, SlPI42, SlPI12, SlPI52, SlPI09, SlPI28, SlPI32*, and *SlPI48*) were increased in resistant tomato plants but decreased in susceptible tomato plants in response to heat stress (Figure 6C).

Under TSWV infection, four *SlPI* genes (*SlPI17, SlPI18, SlPI31*, and *SlPI55*) were upregulated in tomato roots, three genes (*SlPI05, SlPI09* and *SlPI10*) showed no response, and the expressions of the remaining genes were downregulated. In the leaves of tomato, four genes (*SlPI05, SlPI07, SlPI17,* and *SlPI31*) were upregulated, and the expression levels of the remaining genes were downregulated (Figure 6D). The transcriptions of three genes (*SlPI06, SlPI26,* and *SlPI27*) were upregulated in all tested samples due to the wound and the invasion of *Botrytis cinerea* (Figure 6E). In the mature green and ripe red fruits, most of the *SlPI* genes displayed a stronger expression in fruits wound-inoculated with *B. cinerea* than in wounded fruits. Interestingly, eight genes (*SlPI11, SlPI12, SlPI13, SlPI14, SlPI15, SlPI16, SlPI51,* and *SlPI52*) displayed differential expression between the mature green and ripe red fruits.

### 3.6. Expression of SlPI Genes in Different Tomato Tissues

**Figure 7:**
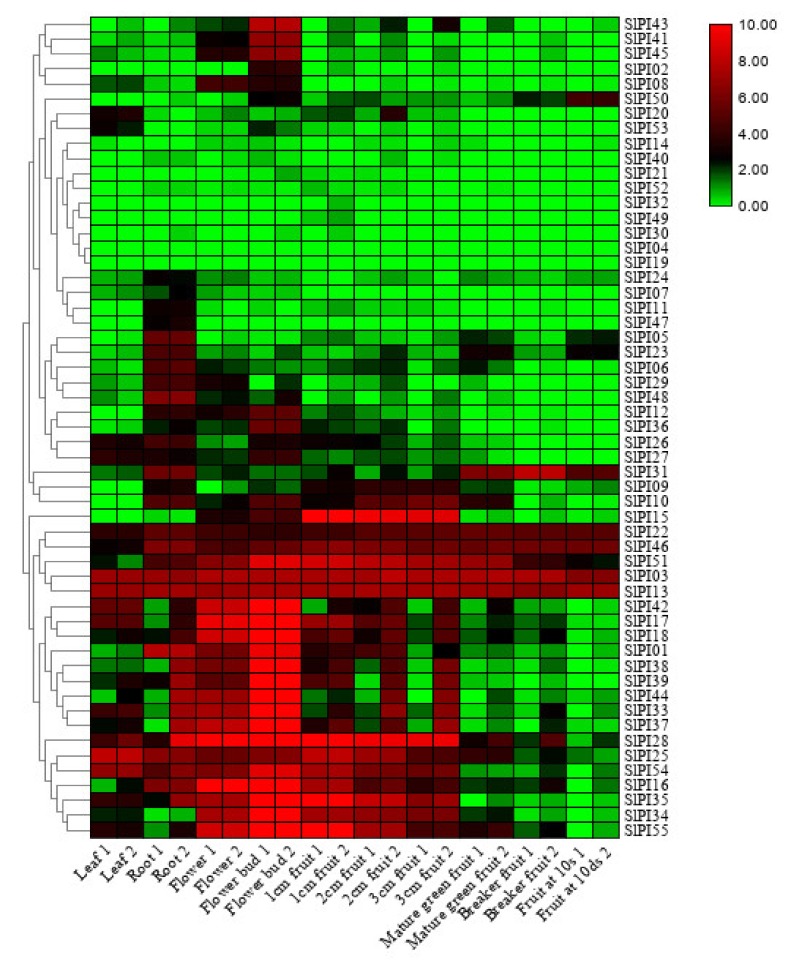
Heat map of the expression patterns of *SlPIs* in 10 tissues/stages. The RNA-seq expression data of 10 tissues were used to reconstruct the expression patterns of *SlPI* genes. The samples were obtained from the leaf, root, flower, flower bud, 1, 2, and 3 cm fruit, mature green fruit, breaker fruit, and fruit at 10 days. Heat map is presented in green/black/red colors that represent low/medium/high expression, respectively.

The expression levels of *SlPI* genes were analyzed in 10 different tissues, including leaf, root, flower, flower bud, 1, 2, and 3 cm fruit, mature green fruit, breaker fruit, and fruit at 10 days, at different developmental stages to obtain their expression patterns in different tissues of tomato and in the developmental stages of the fruit. As shown in Figure 7, some *SlPIs*, including *SlPI03, SlPI13, SlPI22,* and *SlPI46*, were highly expressed in all tissues/stages. By contrast, eight genes, including *SlPI4, SlPI19, SlPI21, SlPI30, SlPI32*, *SlPI*40, *SlPI49,* and *SlPI52*, showed low expression levels in all the tissues/stages. The expression patterns of the other *SlPIs* showed different patterns of temporal and tissue-specific expressions. In addition, 17 genes, including *SlPI01, SlPI16, SlPI17, SlPI18, SlPI25, SlPI28, SlPI33, SlPI34, SlPI45, SlPI37, SlPI38, SlPI39, SlPI41, SlPI42, SlPI44, SlPI54,* and *SlPI55*, were strongly expressed in the flower bud. Specifically, the mRNAs of *SlPI1, SlPI29,* and *SlPI48* accumulated in the root and flower but decreased during fruit development. Moreover, three genes, namely *SlPI28, SlPI34,* and *SlPI35*, showed similar expression patterns with a higher expression at the flower and fruit than the other tissues/stages.

### 3.7. Analysis of PI Gene Expression in Tomato Under Various Hormone Induction and Oxidation

**Figure 8:**
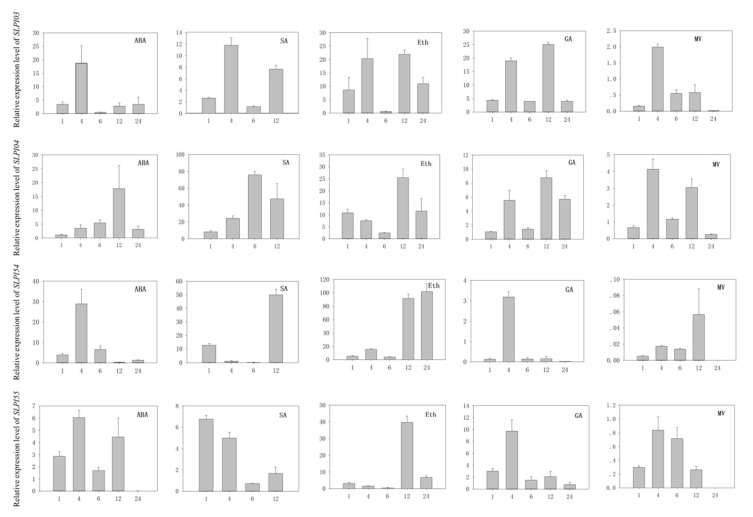
Expression of selected *SlPI* genes under various hormone induction and oxidation. The numbers 1, 4, 6, 12, and 24 indicate the time after treatment (h). At the same time, stress-free plants play a controlling role. Expression of treated plants was compared with that in untreated plants after normalization of values with reference to the tomato *β*-actin gene and is presented as the relative expression level. All samples were collected from three biological replicates of each treatment at specified intervals. Error bars indicate ±SE of the means (*n* = 3). The expression patterns of the selected *SlPI* genes were analyzed by qRT-PCR with gene-specific primers (Supplementary Table S1).

Plant hormones play central roles in plant adaptability under various environments [43], and most abiotic stresses directly or indirectly lead to the rapid accumulation of toxic products, such as free radicals and reactive oxygen species, which cause oxidative stress [44]. To further analyze the function of this gene families, quantitative RT-PCR was used to investigate the transcriptional profiles of PI genes in tomato under ABA, Eth, SA, GA, and oxidative stress (methyl viologen, MV). The transcription levels of PI genes under different treatments were analyzed by qRT-PCR. To avoid the influence of photoperiod, the expression of PI genes was eliminated for 0 h. The expression profiles of all PI genes in tomato were hard to describe. Therefore, four representative members (*SlPI3*, *SlPI4*, *SlPI54*, and *SlPI55*) were assessed from four PI gene families (Figure 1 and Figure 8). 

As shown in Figure 8, *SlPI03* and *SlPI54* decreased immediately after reaching the peak, whereas the expression levels of *SlPI04* and *SlPI55* increased first and then slowly decreased under ABA treatment. Under SA treatment, the expression levels of all three PI genes (*SlPI03*, *SlPI04,* and *SlPI54*) increased, but that of *SlPI55* decreased. Notably, the *SlPI54* levels dramatically increased by approximately 30-fold after 1 h of Eth treatment, which indicated that Eth can promote the expression of *SlPI54*. After GA induction, *SlPI54* and *SlPI55* reached the maximum level at 4 h, whereas *SlPI03* and *SlPI04* were expressed very similarly, with high expression at 4 and 12 h. After 24 h of MV treatment, the expression levels of all four PI genes were almost zero, indicating that MV treatment inhibited the expression of most PI genes. In short, hormones can significantly regulate the expression of PI genes, indicating that the PI gene plays an important role in abiotic response.

## 4. Discussion

### 4.1. Evolution of the SlPI Gene Families

Gene duplication is one of the main forces driving the evolution of the genetic system and genome [45]. The chromosome mapping and the genome distribution of *SlPI* genes indicate that tandem and/or polyploid replication based on the tomato genome annotation database may contribute to the amplification of the *SlPI* genes. In this study, chromosomal localization reveals that 55 PI genes are unevenly distributed on 10 chromosomes of tomato, except for the unclear chromosome mapping of *SlPI01* and *SlPI0*2. Many genes share the same functional domains and similar structure. Therefore, repetitive events have likely contributed to the diversity of the tomato *SlPI* gene families.

In this study, a tandem repeat gene is defined as a gene with an adjacent gene sharing the same domains and no more than one intermediate gene. A total of 29 genes are involved in tandem gene duplication (Figure 3). As shown in Figure 3, three groups of *SlPI* genes (*SlPI 06*/ *SlPI07*/ *SlPI08*/ *SlPI09*/ *SlPI10*, *SlPI11*/ *SlPI12*/ *SlPI13*/ *SlPI14*/*SlPI15*/*SlPI16,* and *SlPI32–SlPI45*) can be identified as tandem duplication genes. These genes belong to the STI, potato inhibitor II, and potato inhibitor I families, respectively, and are located in chromosomes 3 and 9. Hence, tandem duplication has significant contributions to the expansion of the *SlPI* gene families in tomato.

### 4.2. Expression of the SlPI Gene Families

The *SlPI* genes are significantly induced under biotic stresses (*B. cinerea* and TTSWV) (Figure 6D,E), confirming the role of PI in defense against pests and pathogens [26,46,47,48]. Despite substantial efforts in projecting PIs as a valuable weapon against pests, the insect insensitivity and efficient tradeoff tactic has resulted in its commercial failure [49]. However, our study shows that the *SlPI* genes are significantly induced by abiotic stresses, including drought, salt, and heat (Figure 6A–C). These findings, together with our results, led us to explore the PI genes’ role in abiotic stress. 

In the natural environment, plants are constantly challenged by various biological and abiotic stresses. Genes integrate environmental stress signals (such as hormones) when plants encounter adverse ambiance. Plant hormones play a vital role in the ability of plants to encounter abiotic stresses by mediating growth, development, nutrient allocation, and source/sink transitions [43]. The plant hormones ABA, Eth, SA, and GA are involved in diverse plant processes, including the regulation of gene expression during adaptive responses to abiotic and biotic stresses [50]. For example, ABA regulates plant response against several abiotic stresses, such as drought, salt, and cold stresses. Furthermore, ABA plays a negative regulatory function in balancing Arabidopsis resistance response to a necrotrophic fungal pathogen *Plectosphaerella cucumerina*, which naturally colonizes a broad range of Arabidopsis accessions [51,52]. Our results show that the expression of some *SIPIs* can be influenced by ABA, Eth, SA, GA, and MV (Figure 8). The cis-elements of each PI gene promoter have been analyzed to further clarify the relationship between PI gene expression and hormones (Table 2). The ABRE, a cis-element involved in ABA responsiveness, is found in the promoter of 52 PI genes. The TCA-element, a cis-element involved in SA responsiveness, is found in the promoter of 23 PI genes. ARE, a cis-element that is essential for anaerobic induction, is found in 49 *SlPIs*. These conditions are consistent with the expression of PI that can be induced by ABA, SA, and MV (Figure 8). Hence, *SlPIs* may integrate hormone signals to modulate the plant resistance to biotic or abiotic stresses.

A spatiotemporal regulation of the *SlPI* gene families is observed at various stages of tissue development. Two genes (*SlPI3* and *SlPI13*) are highly expressed in all studied tissues, suggesting their possible relationship to the specific housekeeping activities of tomato cells. Moreover, some *SlPI* genes are highly or preferentially expressed, showing tissue- and development-specific expressions (Figure 7). For example, the expression levels of *SlP41, SlP43,* and *SlPI45* in flower bud are higher than those in other tissues. The expression levels of *SlPI25* and *SlPI54* in roots and leaves are higher than those in other tissues. All these genes are expressed preferentially in tissue development and may affect the growth and development of tomatoes (Figure 7). Thus, their functions are worthy of further study. In addition, the expression behaviors of some *SlPI* genes vary in different tissues and stages, implying that *SlPI* proteins may play multiple roles.

### 4.3. Classification and Function of PI Genes in Tomato

In this study, the 55 SlPI proteins of tomato, which contain only one inhibitor domain of PIs, are simple inhibitors. Therefore, they can be easily divided into different families, including Potato inhibitor I, Potato inhibitor II, STI (Kunitz) family, Cysteine proteinase inhibitor, serpin, carboxypeptidase A inhibitor, and peptidase inhibitor I9 families, according to the domain and sequence similarity (Figure 2). These families in PI proteins confer diverse functions, suggesting the multiple roles the PI protein play in plants.

Potato inhibitors I and II are two typical chymotrypsin and trypsin inhibitors [53,54]. Wounding and UV exposure increases the expression of these two inhibitors in tomato and potato leaves [55,56]. Potato PI I and potato PI II are involved in plant defense against herbivorous animals [55]. Among the 55 PI genes of tomato, 22 belong to the potato PI I family (*SlPI04*, *SlPI17*, *SlPI18*, *SlPI30- SlPI45*, and *SlPI47–49*), and 12 belong to the potato PI II family (*SlPI02*, *SlPI11–SlPI16, SlPI29, and SlPI51–54*). In addition, the WUN-motif, a wound response *cis*-element, is found in the promoters of all these PI genes, except *SlPI29*, *SlPI36,* and *SlPI37*. This finding suggests that these PI genes in tomatoes may play an important role in plant wounds and insect resistance.

The Kunitz gene family is a complex family with various PIs and inhibits serine, cysteine, and other hydrolases [57]. The Kunitz PIs reversibly interact with their target proteases to form stable complexes and inhibit their catalytic activity in a competitive or noncompetitive manner [58]. Animal Kunitz PIs are extensively studied and proven to be involved in various physiological processes, such as inflammatory processes [59], thrombosis [60], AIDS [61,62,63], and fungal infections [64]. The family has also been studied in different plants, but most of the work is focused on their defenses in insect attacks because their gene expression levels are upregulated by wounds and insect feeding [65]. Notably, the Kunitz family plays an important role against lepidopteran and coleopteran pests in various plants, including poplar [66], rice [67], tomato [68], potato, and tobacco [69]. In our study, *SlPI06–SlPI10, SlPI26, SlPI27,* and *SlPI55* belong to the Kunitz gene family and may provide defense against herbivores. *SlPI55* is only specifically expressed in flowers and fruits (Figure 6). Hence, *SlPI55* may enhance resistance to biotic stresses at the reproductive stage.

Most plant cysteine PIs, which specifically inhibit the activity of cysteine proteases and are found in plants, insects, and vertebrates, are part of the cystatin superfamily [32]. The cystatin from plants has been classified in a new subfamily (called phytocystatins) due to its special pattern [70]. Although cystatins have been identified in many plant species, only a few cystatins have been well characterized [35]. Some cystatins can prevent the premature proteolytic degradation of newly formed storage proteins during seed development [71,72,73] and reduce the adverse reactions caused by the release of cysteine proteases to the outside of the cells [74]. These PIs also play an important role in plants against herbivorous pests, fungal and nematodes and defense [75,76,77,78,79]. In addition, the overexpression of two cystatins, atCYSa and atCYSb, increases *Arabidopsis* resistance to drought, cold, high salt, and oxidative stresses [80]. Among the 55 PI genes of tomato, *SlPI01*, *SlPI03*, *SlPI05*, *SlPI19*, *SlPI20*, *SlPI25,* and *SlPI46* belong to the family of cysteine PIs and may have similar functions. Notably, *SlPI03* is induced by ABA, SA, Eth, GA, and MV (Figure 7), implying that *SlPI03* plays an important role in tomato resistance to abiotic stress.

The serpin family, the most widely distributed family in PIs (300–500 aa in size), have been identified in viruses, bacteria, plants, microorganisms, and other organisms [9,81,82,83]. Many serpins in animals are involved in the regulation of blood pressure, coagulation, thrombosis, and proteolytic activity associated with inflammatory responses and cell death [84]. However, information about the biological function of serpins in plants is limited. Considering the functional diversity of animal serpins, plant serpins may have a range of functions. Serpins comprise small- to medium-sized gene families comprising 5–10 members [85]. In this study, the tomato serpin superfamily has four members (*SlPI21*, *SlPI22*, *SlPI23,* and *SlPI24*), which is consistent with the small gene family mentioned above. Plant serpins are confounding inhibitors, indicating that serpins can target multiple proteases [86]. The function of serpins is associated with cell death by inhibiting endogenous and exogenous proteases for pest management [87,88]. Serpins are potential signaling molecules involved in the regulation of programmed cell death (PCD) or defense pathways [89]. PCD plays a key role in plant response to stress, including responses to hypoxia, shadowing, extreme temperatures, drought, and oxidative stress. Therefore, four *SlPI* genes (*SlPI21*, *SlPI22*, *SlPI23,* and *SlPI24*) may play important roles in abiotic stress. The understanding of the function of plant serine PIs is far from enough and presents exciting challenges for the future.

The carboxypeptidase A inhibitor family is a family of proteins represented by the potato carboxypeptidase inhibitor (PCI), a metallocarboxypeptidase inhibitor. Previous reports have focused on the response of PCI to insect damage or other mechanical damage [90]. For example, carboxypeptidase inhibitors can prevent pest invasion by hydrolyzing pest digestive proteins [91]. In potato leaves and tomatoes, the carboxypeptidase inhibitor can be induced and used to resist aggressive herbivores [92]. In this study, *SlPI28* belongs to the family of carboxypeptidase A inhibitors, and WUN-motif, a *cis*-element in wound response, is found in the promoter of *SlPI28.* Therefore, *SlPI28* is very likely to be involved in plant defense against herbivores. More recently, two homologs of PCI from tomato (*TCMP-1* and *TCMP-2*) have been shown to be involved in fruit development. Compared with the wild type lines, transgenic lines form flowers earlier and produce fruits earlier [93]. Interestingly, *SlPI28* is poorly expressed before flowering, and its expression increases after flowering, reaching the highest level in flower buds. The spatiotemporal expression pattern of *SlPI28* indicates its role in flower/fruit development. However, a link between the regulation of fruit growth and the inhibitory activity of metallocarboxypeptidases is difficult to determine. Further studies on gene function can provide a better understanding of how genes confer to stress and regulate plant development.

*SlPI50* contains the propeptide domain of the N-terminal peptidase belonging to the subtilisin of the MEROPS family S8A. This domain is also found in members of the MEROPS PI family I9 (peptidase B inhibitor family). This family of PIs is highly specific and inhibits the subtilisin family [94]. The majority of inhibitors of this family, such as the propeptide of subtilisin BPN′ from *Bacillus amyloliquefaciens, Saccharomyces cerevisiae* proteinase B inhibitor 2, and *Pleurotus ostreatus* proteinase A inhibitor 1, are found in bacteria and fungi [95,96]. Recently, subtilisin propeptide-like inhibitor 1 (*SPI-1*) from *Arabidopsis thaliana* has been identified as a member of the I9 inhibitor family [97]. *SPI-1* plays a role in cold stress, senescence, and seed development in *Arabidopsis* [97]. In addition, various hormone-related *cis*-elements, including ABRE (ABA-responsive), CGTCA- and TGACG-motifs (MeJA-responsive), and TGA-element (auxin-responsive), were found in the promoter of *SlPI50*. Hence, *SlPI50* may play an important role in abiotic stresses by integrating hormone signals.

## 5. Conclusions

In this study, the PI gene families were classified and named, and 55 PI genes in the tomato genome were comprehensively analyzed through phylogenetic relationships, gene structure, conserved domains, cis-elements, and expression analysis. *SlPI* genes were characterized by integrating genome organization, comprehensive sequence, conserved domain, gene structure, *cis*-element prediction, and expression profile analysis of 10 different tissues by using RNA-seq and under different stresses (heat, drought, salt, TSWV, and *B. cinereal*) by using microarray atlas. The transcription of some *SlPIs* was induced by hormones, indicating the vital functions of these PI genes in physiological activities. The research results lay the foundation for genetic evolution analysis and functional research. The discovery of new genes related to plant resistance is important for accelerating the cloning of stress resistance genes in tomato.

## Figures and Tables

**Table 1:** Information of the protease inhibitors (PI) gene family in Tomato.

	Identifier	Chromosome Location	AA	PIs/MW	Subcellular Localization
*SlPI01*	Solyc00g071180.2.1	chr00:14605548..14605198	351	5.34/40127.40	cyto: 7, plas: 2, vacu: 2, mito: 1, E.R.: 1, pero: 1
*SlPI02*	Solyc00g145170.1.1	chr00:17251047..17251697	142	6.48/15408.6	extr: 8, nucl: 4, chlo: 1, cyto: 1
*SlPI03*	Solyc01g009020.2.1	chr01:3021936..3024369	113	7.86/12647.4	chlo: 5, cyto: 3, cysk_nucl: 2.5, nucl: 2, mito: 2, extr: 1
*SlPI04*	Solyc01g108380.1.1	chr01:95700909..95701233	77	8.45/8071.5	chlo: 11, extr: 2, nucl: 1
*SlPI05*	Solyc03g097270.2.1	chr03:59587548..59593766	235	6.39/26249.79	vacu: 6, extr: 3, chlo: 2, cyto: 1, mito: 1, golg: 1
*SlPI06*	Solyc03g098670.1.1	chr03:60963114..60963719	113	7.86/12647.4	extr: 9, vacu: 3, chlo: 1, cyto: 1
*SlPI07*	Solyc03g098700.1.1	chr03:60981171..60981830	219	5.07/24278.1	extr: 10, chlo: 3, E.R.: 1
*SlPI08*	Solyc03g098710.1.1	chr03:60989342..60990016	224	6.50/24726.1	extr: 5, vacu: 3, E.R.: 2, chlo: 1, cyto: 1, mito: 1, plas: 1
*SlPI09*	Solyc03g098780.1.1	chr03:61061419..61062084	221	7.48/24500.2	extr: 7, vacu: 3, cyto: 1, mito: 1, plas: 1, golg: 1
*SlPI10*	Solyc03g098790.1.1	chr03:61068935..61069598	220	8.84/24188.08	extr: 7, vacu: 3, chlo: 2, cyto: 1, mito: 1
*SlPI11*	Solyc03g020080.2.1	chr03:61158441..61159659	159	7.85/17304.9	extr: 8, chlo: 2, nucl: 2, cyto: 1, vacu: 1v
*SlPI12*	Solyc03g020070.2.1	chr03:61164764..61165829	159	8.12/17144.7	extr: 11, chlo: 2, cyto: 1
*SlPI13*	Solyc03g020060.2.1	chr03:61171088..61172165	206	7.31/22018.4	extr: 6, nucl: 4, chlo: 2, cyto: 1, vacu: 1
*SlPI14*	Solyc03g020050.2.1	chr03:61174085..61175022	201	4.90/21370.2	extr: 12, golg: 2
*SlPI15*	Solyc03g020040.2.1	chr03:61180880..61181788	207	8.53/22682.2	extr: 11, cyto: 1, vacu: 1, E.R.: 1
*SlPI16*	Solyc03g020030.2.1	chr03:61189044..61189951	207	8.60/22653.2	extr: 11, cyto: 1, vacu: 1, E.R.: 1
*SlPI17*	Solyc03g020010.1.1	chr03:61222484..61223101	205	4.83/22945.4	extr:7, vacu: 2, golg: 2, chlo: 1, cyto: 1, plas: 1
*SlPI18*	Solyc03g019690.1.1	chr03:61452670..61453323	217	6.80/23718.9	extr: 8, chlo: 4, cyto: 1, vacu: 1
*SlPI19*	Solyc04g014780.1.1	chr04:5029049..5029411	120	6.28/13556.5	extr: 9, vacu: 2, chlo: 1, cyto: 1, plas: 1
*SlPI20*	Solyc04g014790.1.1	chr04:5030604..5031014	136	8.56/15121.3	cyto: 5, extr: 5, chlo: 2, nucl: 1, E.R._vacu: 1
*SlPI21*	Solyc04g079370.2.1	chr04:63894231..63896110	390	5.25/44417.73	cyto: 6, cysk: 5, chlo: 2, nucl: 1
*SlPI22*	Solyc04g079440.2.1	chr04:63943215..63945697	391	5.41/42832.81	cyto: 2, vacu: 2, plas: 1.5, chlo: 1, nucl: 1, mito: 1, golg: 1
*SlPI23*	Solyc04g079470.2.1	chr04:63948225..63950136	390	5.53/43200.55	cyto: 4, vacu: 3, E.R.: 3, nucl: 2, chlo: 1, plas: 1
*SlPI24*	Solyc04g079480.2.1	chr04:63950580..63952328	416	8.19/46153.11	chlo: 9, mito: 2, extr: 2, nucl: 1
*SlPI25*	Solyc05g054120.1.1	chr05:64086541..64086930	603	6.23/68743.2	chlo: 12, extr: 1, E.R._vacu: 1
*SlPI26*	Solyc06g072220.1.1	chr06:44506328..44506963	211	9.31/23333.3	extr: 6, vacu: 4, mito: 2, chlo: 1, golg: 1
*SlPI27*	Solyc06g072230.1.1	chr06:44509088..44509690	200	8.85/22259.8	extr: 7, vacu: 3, mito: 2, chlo: 1, cyto: 1
*SlPI28*	Solyc07g007250.2.1	chr07:1989153..1990888	77	6.49/8353.7	extr: 13, vacu: 1
*SlPI29*	Solyc07g054720.1.1	chr07:62945748..62946391	77	7.46/8343.6	extr: 10, vacu: 3, cyto: 1
*SlPI30*	Solyc08g080020.1.1	chr08:63378916..63379230	104	4.64/11425.3	extr: 10, vacu: 2, chlo: 1, cyto: 1
*SlPI31*	Solyc08g080630.2.1	chr08:63880194..63882176	119	5.33/13160.3	cyto: 5, extr: 3, E.R.: 2, golg: 2, nucl: 1, vacu: 1
*SlPI32*	Solyc09g083430.1.1	chr09:69089700..69089987	71	5.17/8066.5	cyto_nucl: 5.5, cyto: 5, chlo: 2, extr: 2, golg: 2, E.R.: 1
*SlPI33*	Solyc09g083440.2.1	chr09:69093760..69095168	111	6.58/12359.7	extr: 10, cyto: 2, chlo: 1, cysk: 1
*SlPI34*	Solyc09g084440.2.1	chr09:69112518..69113780	111	5.80/12483.7	extr: 7, cyto: 3, chlo: 2, vacu: 1, E.R.: 1
*SlPI35*	Solyc09g084450.2.1	chr09:69123240..69124920	111	7.76/12580.8	extr: 8, vacu: 2, chlo: 1, cyto: 1, mito: 1, E.R.: 1
*SlPI36*	Solyc09g084460.2.1	chr09:69127703..69129440	111	5.82/12656.9	extr: 6, vacu: 4, chlo: 3, nucl: 1
*SlPI37*	Solyc09g084470.2.1	chr09:69132578..69133986	111	4.69/12584.8	extr: 10, chlo: 1, nucl: 1, cysk: 1, golg: 1
*SlPI38*	Solyc09g084480.2.1	chr09:69137050..69138791	111	6.58/12557.8	extr: 6, cyto: 4, vacu: 2, chlo: 1, mito: 1
*SlPI39*	Solyc09g084490.2.1	chr09:69140543..69142228	110	6.57/12366.6	extr: 10, cyto: 2, nucl: 1, cysk: 1
*SlPI40*	Solyc09g089490.2.1	chr09:69207347..69208204	117	6.82/13045.4	extr: 8, vacu: 3, chlo: 1, cyto: 1, plas: 1
*SlPI41*	Solyc09g089500.2.1	chr09:69211165..69212219	112	5.76/12668.9	extr: 9, vacu: 2, chlo: 1, cyto: 1, mito: 1
*SlPI42*	Solyc09g089510.2.1	chr09:69218803..69219857	117	8.64/13121.6	extr: 10, chlo: 1, cyto: 1, mito: 1, vacu: 1
*SlPI43*	Solyc09g089520.2.1	chr09:69225548..69226271	116	9.00/12977.4	extr: 4, vacu: 4, cyto: 2, chlo: 1, nucl: 1, E.R.: 1, golg: 1
*SlPI44*	Solyc09g089530.2.1	chr09:69229994..69233425	114	6.82/12715.9	cyto: 5, extr: 5, chlo: 1, nucl: 1, mito: 1, cysk: 1
*SlPI45*	Solyc09g089540.2.1	chr09:69238075..69239765	114	5.66/12700.9	extr: 9, chlo: 1, cyto: 1, mito: 1, vacu: 1, E.R.: 1
*SlPI46*	Solyc09g097850.1.1	chr09:71842796..71843200	134	9.78/14721.1	chlo: 9, cyto: 2, extr: 2, nucl: 1
*SlPI47*	Solyc10g086090.1.1	chr10:65046063..65046671	95	10.11/10682.7	extr: 8, vacu: 2, chlo: 1, nucl: 1, cyto: 1, plas: 1
*SlPI48*	Solyc10g086100.1.1	chr10:65049349..65050364	94	9.38/10521.4	extr: 5, vacu: 5, golg: 2, chlo: 1, nucl: 1
*SlPI49*	Solyc11g007050.1.1	chr11:1528822..1529124	100	7.59/10955.9	extr: 9, vacu: 2, cyto: 1, plas: 1, golg: 1
*SlPI50*	Solyc11g018590.1.1	chr11:8761757..8762969	102	6.83/11488.4	extr: 11, chlo: 1, cyto: 1, vacu: 1
*SlPI51*	Solyc11g020960.1.1	chr11:12996643..12997161	146	8.58/15636.8	extr: 13, vacu: 1
*SlPI52*	Solyc11g020990.1.1	chr11:13167991..13169189	360	7.55/39279.7	extr: 9, chlo: 1, cyto: 1, plas: 1, vacu: 1, E.R.: 1
*SlPI53*	Solyc11g021020.1.1	chr11:13195204..13195581	125	5.20/13985.9	chlo: 5, mito: 4, extr: 4, golg: 1
*SlPI54*	Solyc11g021060.1.1	chr11:13312553..13313384	223	5.32/24697.3	extr: 11, vacu: 2, chlo: 1
*SlPI55*	Solyc11g022590.1.1	chr11:14564635..14565312	225	9.37/25188.2	extr: 6, vacu: 3, chlo: 2, cyto: 1, mito: 1, golg: 1

The subcellular location of tomato PI proteins were predicted using WoLF PSORT (https://wolfpsort.hgc.jp/) AA number of amino acid, pIs theoretical isoelectric point, MW molecular weight (KDa), nucl nucleus, mito mitochondria, chlo chloroplast, cyto cytosol, E.R. endoplasmic reticulum, Plas plasma membrane, extr extracellular, pero peroxisome, golg golgi apparatus, vacu vacuole, Testk used for kNN is: 14.

**Table 2:** Functionally annotated cis-elements identified in the promoters of more than 20 *SlPIs.*

*Cis*-Element	Number of Genes	Functions of *Cis*-Elements
Box 4	55	part of a conserved DNA module involved in light responsiveness
CAAT-box	55	common cis-acting element in promoter and enhancer regions
TATA-box	55	core promoter element around -30 of transcription start
ABRE	52	cis-acting element involved in the abscisic acid responsiveness
G-box	50	cis-acting regulatory element involved in light responsiveness
ARE	48	cis-acting regulatory element essential for the anaerobic induction
G-Box	44	cis-acting regulatory element involved in light responsiveness
TCT-motif	41	part of a light responsive element
WUN-motif	42	wound-responsive element
GT1-motif	38	light responsive element
CGTCA-motif	38	cis-acting regulatory element involved in the MeJA-responsiveness
TGACG-motif	38	cis-acting regulatory element involved in the MeJA-responsiveness
GATA-motif	29	part of a light responsive element
I-box	27	part of a light responsive element
TC-rich repeats	25	cis-acting element involved in defense and stress responsiveness
chs-CMA1a	25	part of a light responsive element
TCA-element	23	cis-acting element involved in salicylic acid responsiveness
MBS	23	MYB binding site involved in drought-inducibility
LTR	21	cis-acting element involved in low-temperature responsiveness
TGA-element	20	auxin-responsive element
CAT-box	20	cis-acting regulatory element related to meristem expression

## Supplementary Materials

The following are available online at https://www.mdpi.com/2073-4425/11/1/1/s1, Figure S1: Amino acid sequence comparison of *SlPI* genes, Table S1: List of primers used for RT-PCR, Table S2: The information of the conserved domains, Table S3: Cis-elements identified in the promoters of *SlPI* genes, Table S4: Cis-elements identified in the promoters of more than 20 *SlPI* genes, Supplementary Table S5: Information on probe sets used for microarray expression analysis.

## References

[1] Brix K., Stöcker W. (2013). Proteases: Structure and Function.

[2] Di Cera E. (2009). Serine proteases. IUBMB Life.

[3] Lingaraju M., Gowda L.R. (2008). A Kunitz trypsin inhibitor of Entada scandens seeds: Another member with single disulfide bridge. BBA Proteins Proteom..

[4] Rehman S., Aziz E., Akhtar W., Ilyas M., Mahmood T. (2017). Structural and functional characteristics of plant proteinase inhibitor-II (PI-II) family. Biotechnol. Lett..

[5] De Leo F., Volpicella M., Licciulli F., Liuni S., Gallerani R., Ceci L.R. (2002). PLANT-PIs: A database for plant protease inhibitors and their genes. Nucl. Acids Res..

[6] Tamhane V.A., Giri A.P., Kumar P., Gupta V.S. (2009). Spatial and temporal expression patterns of diverse Pin-II proteinase inhibitor genes in *Capsicum annuum* Linn. Gene.

[7] Lawrence P.K., Koundal K.R. (2002). Plant protease inhibitors in control of phytophagous insects. Electron. J. Biotechnol..

[8] Birk Y. (2003). Plant Protease Inhibitors: Significance in Nutrition, Plant Protection, Cancer Prevention and Genetic Engineering.

[9] Rawlings N.D., Tolle D.P., Barrett A.J. (2004). Evolutionary families of peptidase inhibitors. Biochem. J..

[10] Rawlings N.D., Barrett A.J., Thomas P.D., Huang X., Bateman A., Finn R.D. (2017). The MEROPS database of proteolytic enzymes, their substrates and inhibitors in 2017 and a comparison with peptidases in the PANTHER database. Nucl. Acids Res..

[11] Ryan C.A. (1990). Protease inhibitors in plants: Genes for improving defenses against insects and pathogens. Annu. Rev. Phytopathol..

[12] Broadway R.M. (1995). Are insects resistant to plant proteinase inhibitors?. J. Insect Physiol..

[13] Jongsma M.A., Bolter C. (1997). The adaptation of insects to plant protease inhibitors. J. Insect Physiol..

[14] Zavala J.A., Patankar A.G., Gase K., Hui D., Baldwin I.T. (2004). Manipulation of endogenous trypsin proteinase inhibitor production in Nicotiana attenuata demonstrates their function as antiherbivore defenses. Plant Physiol..

[15] Green T., Ryan C.A. (1972). Wound-induced proteinase inhibitor in plant leaves: A possible defense mechanism against insects. Science.

[16] Hilder V.A., Gatehouse A.M., Sheerman S.E., Barker R.F., Boulter D. (1987). A novel mechanism of insect resistance engineered into tobacco. Nature.

[17] Chen P.J., Senthilkumar R., Jane W.N., He Y., Tian Z., Yeh K.W. (2014). Transplastomic Nicotiana benthamiana plants expressing multiple defence genes encoding protease inhibitors and chitinase display broad-spectrum resistance against insects, pathogens and abiotic stresses. Plant Biotech. J..

[18] Telang M.A., Giri A.P., Pyati P.S., Gupta V.S., Tegeder M., Franceschi V.R. (2009). Winged bean chymotrypsin inhibitors retard growth of *Helicoverpa armigera*. Gene.

[19] Abdeen A., Virgós A., Olivella E., Villanueva J., Avilés X., Gabarra R., Prat S. (2005). Multiple insect resistance in transgenic tomato plants over-expressing two families of plant proteinase inhibitors. Plant Mol. Biol..

[20] Broadway R.M., Duffey S.S. (1986). Plant proteinase inhibitors: Mechanism of action and effect on the growth and digestive physiology of larval Heliothis zea and Spodoptera exiqua. J. Insect Physiol..

[21] Duan X., Li X., Xue Q., Abo-EI-Saad M., Xu D., Wu R. (1996). Transgenic rice plants harboring an introduced potato proteinase inhibitor II gene are insect resistant. Nat. Biotech..

[22] Gatehouse A.M., Davison G.M., Newell C.A., Merryweather A., Hamilton W.D., Burgess E.P., Gilbert R.J., Gatehouse J.A. (1997). Transgenic potato plants with enhanced resistance to the tomato moth, Lacanobia oleracea: Growth room trials. Mol. Breed..

[23] Srinivasan A., Giri A.P., Harsulkar A.M., Gatehouse J.A., Gupta V.S. (2005). A Kunitz trypsin inhibitor from chickpea (*Cicer arietinum* L.) that exerts anti-metabolic effect on podborer (*Helicoverpa armigera*) larvae. Plant Mol. Biol..

[24] Johnson R., Narvaez J., An G., Ryan C. (1989). Expression of proteinase inhibitors I and II in transgenic tobacco plants: Effects on natural defense against Manduca sexta larvae. Proc. Natl. Acad. Sci. USA.

[25] Urwin P.E., Atkinson H.J., Waller D.A., McPherson M.J. (1995). Engineered oryzacystatin-I expressed in transgenic hairy roots confers resistance to *Globodera pallida*. Plant J..

[26] Gatehouse J.A. (2011). Prospects for using proteinase inhibitors to protect transgenic plants against attack by herbivorous insects. Curr. Protein Pept. Sci..

[27] Cloutier C., Jean C., Fournier M., Yelle S., Michaud D. (2000). Adult Colorado potato beetles, Leptinotarsa decemlineata compensate for nutritional stress on oryzacystatin I-transgenic potato plants by hypertrophic behavior and over-production of insensitive proteases. Arch. Insect Biochem. Physiol..

[28] De Leo F., Bonadé-Bottino M.A., Ceci L.R., Gallerani R., Jouanin L. (1998). Opposite effects on Spodoptera littoralis larvae of high expression level of a trypsin proteinase inhibitor in transgenic plants. Plant Physiol..

[29] Gosti F., Bertauche N., Vartanian N., Giraudat J. (1995). Abscisic acid-dependent and-independent regulation of gene expression by progressive drought in Arabidopsis thaliana. Mol. Gen. Genet. MGG.

[30] Kim S., Hong Y.-N., An C.S., Lee K.-W. (2001). Expression characteristics of serine proteinase inhibitor II under variable environmental stresses in hot pepper (*Capsicum annuum* L.). Plant Sci..

[31] Pernas M., Sánchez-Monge R., Salcedo G. (2000). Biotic and abiotic stress can induce cystatin expression in chestnut. Febs Lett..

[32] Gaddour K., Vicente-Carbajosa J., Lara P., Isabel-Lamoneda I., Díaz I., Carbonero P. (2001). A constitutive cystatin-encoding gene from barley (Icy) responds differentially to abiotic stimuli. Plant Mol. Biol..

[33] Huang Y., Xiao B., Xiong L. (2007). Characterization of a stress responsive proteinase inhibitor gene with positive effect in improving drought resistance in rice. Planta.

[34] Srinivasan T., Kumar K.R.R., Kirti P.B. (2009). Constitutive expression of a trypsin protease inhibitor confers multiple stress tolerance in transgenic tobacco. Plant Cell Physiol..

[35] Chen G.-Q., Zhang D., Shen X.-H. (2018). Cloning and characterization of ApCystatin, a plant cystatin gene from Agapanthus praecox ssp. orientalis responds to abiotic stress. Protein Expr. Purif..

[36] Titarenko E., Rojo E., Leon J., Sanchez-Serrano J.J. (1997). Jasmonic acid-dependent and-independent signaling pathways control wound-induced gene activation in *Arabidopsis thaliana*. Plant Physiol..

[37] Koiwa H., Bressan R.A., Hasegawa P.M. (1997). Regulation of protease inhibitors and plant defense. Trends Plant Sci..

[38] Morris K., Mackerness S.A.H., Page T., John C.F., Murphy A.M., Carr J.P., Buchanan-Wollaston V. (2000). Salicylic acid has a role in regulating gene expression during leaf senescence. Plant J..

[39] Li J., Su X., Wang Y., Yang W., Pan Y., Su C., Zhang X. (2018). Genome-wide identification and expression analysis of the BTB domain-containing protein gene family in tomato. Genes Genom..

[40] Tamura K., Stecher G., Peterson D., Filipski A., Kumar S. (2013). MEGA6: Molecular evolutionary genetics analysis version 6.0. Mol. Biol. Evol..

[41] Hu B., Jin J., Guo A., Zhang H., Luo J., Gao G. (2015). GSDS 2.0: An upgraded gene feature visualization server. Bioinformatics.

[42] Bostan H., Chiusano M.L. (2015). NexGenEx-Tom: A gene expression platform to investigate the functionalities of the tomato genome. BMC Plant Biol..

[43] Peleg Z., Blumwald E. (2011). Hormone balance and abiotic stress tolerance in crop plants. Curr. Opin. Plant Biol..

[44] Oberschall A., Deák M., Török K., Sass L., Vass I., Kovács I., Fehér A., Dudits D., Horváth G.V. (2000). A novel aldose/aldehyde reductase protects transgenic plants against lipid peroxidation under chemical and drought stresses. Plant J..

[45] Moore R.C., Purugganan M.D. (2003). The early stages of duplicate gene evolution. Proc. Natl. Acad. Sci. USA.

[46] Joshi B.N., Sainani M.N., Bastawade K.B., Gupta V.S., Ranjekar P.K. (1998). Cysteine protease inhibitor from pearl millet: A new class of antifungal protein. Biochem. Biophys. Res. Commun..

[47] Dunaevsky Y.E., Gladysheva I.P., Pavlukova E.B., Beliakova G.A., Gladyshev D.P., Papisova A.I., Larionova N.I., Belozersky M.A. (1997). The anionic protease inhibitor BWI-1 from buckwheat seeds. Kinetic properties and possible biological role. Physiol. Plant..

[48] Broadway R.M. (1996). Dietary proteinase inhibitors alter complement of midgut proteases. Arch. Insect Biochem. Physiol..

[49] Singh S., Singh A., Kumar S., Mittal P., Singh I.K. (2018). Protease inhibitors: Recent advancement in its usage as a potential biocontrol agent for insect pest management. Insect Sci..

[50] Anderson J.P., Badruzsaufari E., Schenk P.M., Manners J.M., Desmond O.J., Ehlert C., Maclean D.J., Ebert P.R., Kazan K. (2004). Antagonistic interaction between abscisic acid and jasmonate-ethylene signaling pathways modulates defense gene expression and disease resistance in Arabidopsis. Plant Cell.

[51] Llorente F., Alonso-Blanco C., Sánchez-Rodriguez C., Jorda L., Molina A. (2005). ERECTA receptor-like kinase and heterotrimeric G protein from Arabidopsis are required for resistance to the necrotrophic fungus Plectosphaerella cucumerina. Plant J..

[52] Sánchez-Vallet A., López G., Ramos B., Delgado-Cerezo M., Riviere M.-P., Llorente F., Fernández P.V., Miedes E., Estevez J.M., Grant M. (2012). Disruption of abscisic acid signaling constitutively activates Arabidopsis resistance to the necrotrophic fungus Plectosphaerella cucumerina. Plant Physiol..

[53] Melville J.C., Ryan C.A. (1972). Chymotrypsin Inhibitor I from Potatoes Large Scale Preparation and Characterization of its Subunit Components. J. Biol. Chem..

[54] Bryant J., Green T.R., Gurusaddaiah T., Ryan C.A. (1976). Proteinase inhibitor II from potatoes: Isolation and characterization of its protomer components. Biochemistry.

[55] Bergey D.R., Howe G.A., Ryan C.A. (1996). Polypeptide signaling for plant defensive genes exhibits analogies to defense signaling in animals. Proc. Natl. Acad. Sci. USA.

[56] Conconi A., Smerdon M.J., Howe G.A., Ryan C.A. (1996). The octadecanoid signalling pathway in plants mediates a response to ultraviolet radiation. Nature.

[57] Oliva M.L.V., Silva M.C., Sallai R.C., Brito M.V., Sampaio M.U. (2010). A novel subclassification for Kunitz proteinase inhibitors from leguminous seeds. Biochimie.

[58] Rustgi S., Boex-Fontvieille E., Reinbothe C., von Wettstein D., Reinbothe S. (2018). The complex world of plant protease inhibitors: Insights into a Kunitz-type cysteine protease inhibitor of Arabidopsis thaliana. Commun. Integr. Biol..

[59] Mello G.C., Desouza I.A., Marangoni S., Novello J.C., Antunes E., Macedo M.L.R. (2006). Oedematogenic activity induced by Kunitz-type inhibitors from Dimorphandra mollis seeds. Toxicon.

[60] Oliva M.L.V., Sampaio M.U. (2008). Bauhinia Kunitz-type proteinase inhibitors: Structural characteristics and biological properties. Biol. Chem..

[61] Cheung A.H., Wong J.H., Ng T. (2009). Trypsin-chymotrypsin inhibitors from Vigna mungo seeds. Protein Pept. Lett..

[62] Fang E.F., Wong J.H., Ng T.B. (2010). Thermostable Kunitz trypsin inhibitor with cytokine inducing, antitumor and HIV-1 reverse transcriptase inhibitory activities from Korean large black soybeans. J. Biosci. Bioeng..

[63] Asztalos B.F., Schaefer E.J., Horvath K.V., Cox C.E., Skinner S., Gerrior J., Gorbach S.L., Wanke C. (2006). Protease inhibitor-based HAART, HDL, and CHD-risk in HIV-infected patients. Atherosclerosis.

[64] Bhattacharyya A., Babu C.R. (2009). Purification and biochemical characterization of a serine proteinase inhibitor from *Derris trifoliata* Lour. seeds: Insight into structural and antimalarial features. Phytochemistry.

[65] Arnaiz A., Talavera-Mateo L., González-Melendi De León P., Martinez M., Diaz I., Santamaria M.E. (2018). Arabidopsis Kunitz Trypsin Inhibitors in Defence Against Spider Mites. Front. Plant Sci..

[66] Confalonieri M., Allegro G., Balestrazzi A., Fogher C., Delledonne M. (1998). Regeneration of *Populus nigra* transgenic plants expressing a Kunitz proteinase inhibitor (KTi 3) gene. Mol. Breed..

[67] Lee S.I., Lee S.-H., Koo J.C., Chun H.J., Lim C.O., Mun J.H., Song Y.H., Cho M.J. (1999). Soybean Kunitz trypsin inhibitor (SKTI) confers resistance to the brown planthopper (Nilaparvata lugens Stål) in transgenic rice. Mol. Breed..

[68] Gatehouse A.M., Norton E., Davison G.M., Babbé S.M., Newell C.A., Gatehouse J.A. (1999). Digestive proteolytic activity in larvae of tomato moth, Lacanobia oleracea; effects of plant protease inhibitors in vitro and in vivo. J. Insect Physiol..

[69] Marchetti S., Delledonne M., Fogher C., Chiaba C., Chiesa F., Savazzini F., Giordano A. (2000). Soybean Kunitz, C-II and PI-IV inhibitor genes confer different levels of insect resistance to tobacco and potato transgenic plants. Theor. Appl. Genet..

[70] Aceituno-Valenzuela U., Covarrubias M.P., Aguayo M.F., Valenzuela-Riffo F., Espinoza A., Gaete-Eastman C., Herrera R., Handford M., Norambuena L. (2018). Identification of a type II cystatin in Fragaria chiloensis: A proteinase inhibitor differentially regulated during achene development and in response to biotic stress-related stimuli. Plant Physiol. Biochem..

[71] Arai S., Matsumoto I., Emori Y., Abe K. (2002). Plant seed cystatins and their target enzymes of endogenous and exogenous origin. J. Agric. Food Chem..

[72] Diaz-Mendoza M., Dominguez-Figueroa J.D., Velasco-Arroyo B., Cambra I., Gonzalez-Melendi P., Lopez-Gonzalvez A., Garcia A., Hensel G., Kumlehn J., Diaz I. (2016). HvPap-1 C1A Protease and HvCPI-2 Cystatin Contribute to Barley Grain Filling and Germination. Plant Physiol..

[73] Abe K., Emori Y., Kondo H., Suzuki K., Arai S. (1987). Molecular-Cloning of a Cysteine Proteinase-Inhibitor of Rice (Oryzacystatin)—Homology with Animal Cystatins and Transient Expression in the Ripening Process of Rice Seeds. J. Biol. Chem..

[74] Belenghi B., Acconcia F., Trovato M., Perazzolli M., Bocedi A., Polticelli F., Ascenzi P., Delledonne M. (2003). AtCYS1, a cystatin from Arabidopsis thaliana, suppresses hypersensitive cell death. Eur. J. Biochem..

[75] Carrillo L., Martinez M., Ramessar K., Cambra I., Castañera P., Ortego F., Díaz I. (2011). Expression of a barley cystatin gene in maize enhances resistance against phytophagous mites by altering their cysteine-proteases. Plant Cell Rep..

[76] Wang K.M., Kumar S., Cheng Y.S., Venkatagiri S., Yang A.H., Yeh K.W. (2008). Characterization of inhibitory mechanism and antifungal activity between group-1 and group-2 phytocystatins from taro (*Colocasia esculenta*). FEBS J..

[77] Lilley C., Urwin P., McPherson M., Atkinson H. (1996). Characterization of intestinally active proteinases of cystnematodes. Parasitology.

[78] Urwin P.E., Lilley C.J., McPherson M.J., Atkinson H.J. (1997). Resistance to both cyst and root-knot nematodes conferred by transgenic Arabidopsis expressing a modified plant cystatin. Plant J..

[79] Yu Y., Zhang G., Li Z., Cheng Y., Gao C., Zeng L., Chen J., Yan L., Sun X., Guo L. (2017). Molecular Cloning, Recombinant Expression and Antifungal Activity of BnCPI, a Cystatin in Ramie (*Boehmeria nivea* L.). Genes.

[80] Zhang X., Liu S., Takano T. (2008). Two cysteine proteinase inhibitors from Arabidopsis thaliana, AtCYSa and AtCYSb, increasing the salt, drought, oxidation and cold tolerance. Plant Mol. Biol..

[81] Irving J.A., Pike R.N., Lesk A.M., Whisstock J.C. (2000). Phylogeny of the serpin superfamily: Implications of patterns of amino acid conservation for structure and function. Genome Res..

[82] Law R.H., Zhang Q., McGowan S., Buckle A.M., Silverman G.A., Wong W., Rosado C.J., Langendorf C.G., Pike R.N., Bird P.I. (2006). An overview of the serpin superfamily. Genome Biol..

[83] Gettins P.G. (2002). Serpin structure, mechanism, and function. Chem. Rev..

[84] Silverman G.A., Whisstock J.C., Bottomley S.P., Huntington J.A., Kaiserman D., Luke C.J., Pak S.C., Reichhart J.-M., Bird P.I. (2010). Serpins flex their muscle I. Putting the clamps on proteolysis in diverse biological systems. J. Biol. Chem..

[85] Cohen M., Davydov O., Fluhr R. (2019). Plant serpin protease inhibitors: Specificity and duality of function. J. Exp. Bot..

[86] Grosse-Holz F.M., van der Hoorn R.A. (2016). Juggling jobs: Roles and mechanisms of multifunctional protease inhibitors in plants. New Phytol..

[87] Fluhr R., Lampl N., Roberts T.H. (2012). Serpin protease inhibitors in plant biology. Physiol. Plant..

[88] Jamal F., Pandey P.K., Singh D., Khan M. (2013). Serine protease inhibitors in plants: Nature’s arsenal crafted for insect predators. Phytochem. Rev..

[89] Roberts T.H., Hejgaard J. (2008). Serpins in plants and green algae. Funct. Integr. Genom..

[90] Ryan C.A. (2000). The systemin signaling pathway: Differential activation of plant defensive genes. Biochim. Biophys. Acta.

[91] Hass G.M., Ryan C.A. (1981). Carboxypeptidase Inhibitor from Potatoes. Methods in Enzymology.

[92] Díez-Díaz M., Conejero V., Rodrigo I., Pearce G., Ryan C.A. (2004). Isolation and characterization of wound-inducible carboxypeptidase inhibitor from tomato leaves. Phytochemistry.

[93] Molesini B., Rotino G., Dusi V., Chignola R., Sala T., Mennella G., Francese G., Pandolfini T. (2018). Two metallocarboxypeptidase inhibitors are implicated in tomato fruit development and regulated by the Inner No Outer transcription factor. Plant Sci..

[94] Harish B., Uppuluri K.B. (2018). Microbial serine protease inhibitors and their therapeutic applications. Int. J. Biol. Macromol..

[95] Maier K., Müller H., Holzer H. (1979). Purification and molecular characterization of two inhibitors of yeast proteinase B. J. Biol. Chem..

[96] Dohmae N., Takio K., Tsumuraya Y., Hashimoto Y. (1995). The complete amino acid sequences of two serine proteinase inhibitors from the fruiting bodies of a basidiomycete, Pleurotus ostreatus. Arch. Biochem. Biophys..

[97] Hohl M., Stintzi A., Schaller A. (2017). A novel subtilase inhibitor in plants shows structural and functional similarities to protease propeptides. J. Biol. Chem..

