# Supplementary material for: Genome-Wide Identification and Expression Analysis of the Protease Inhibitor Gene Families in Tomato
Source: Genes (Basel). 2019 Dec 18;11(1):1. doi: 10.3390/genes11010001 (PMC7017114; doi:10.3390/genes11010001)
Supplement: Supplementary file 1 [file genes-11-00001-s001.zip › Supplementary Files/Supplementary Figure S1. Amino acid sequence comparison of SlPI genes..pdf]

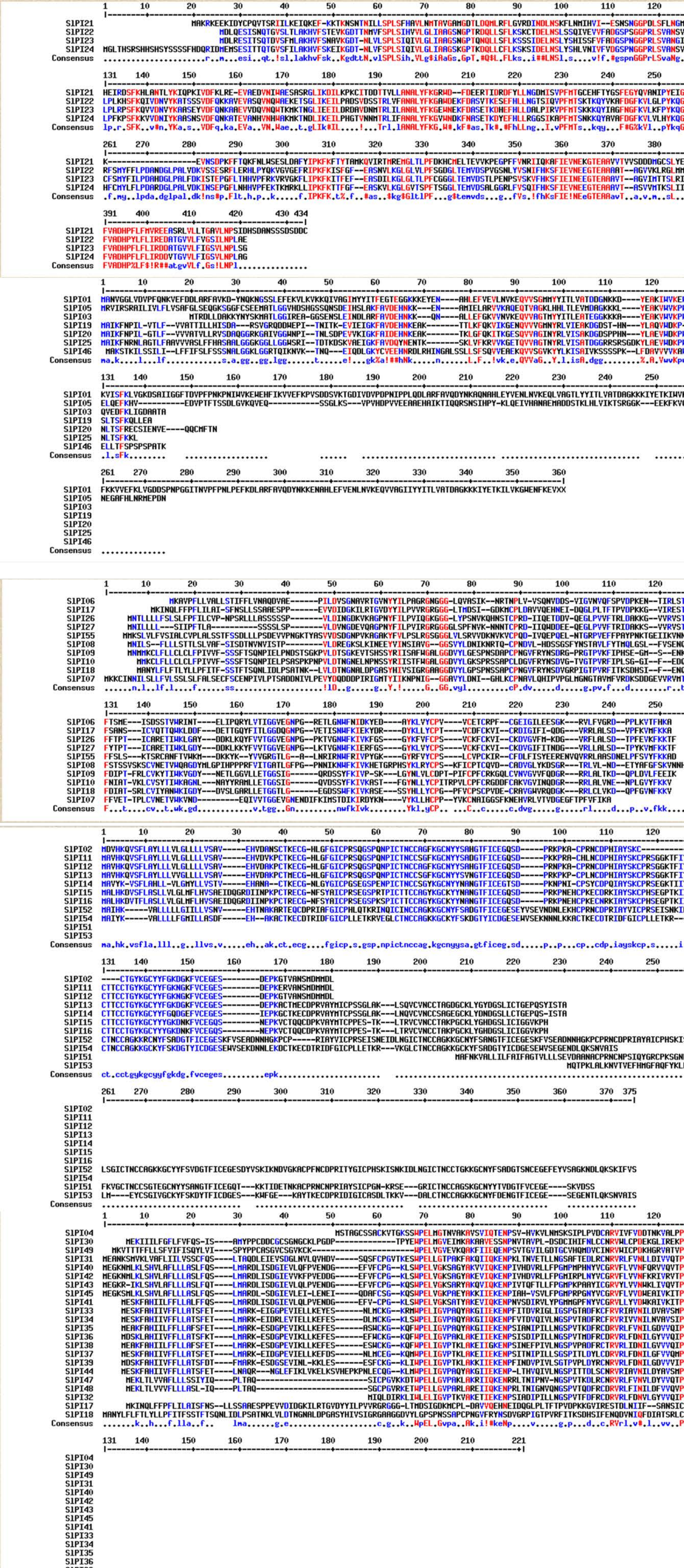
SlPI21
SlPI22
SlPI23
SlPI24
Consensus
SlPI01
SlPI05
SlPI03
SlPI19
SlPI20
SlPI25
SlPI46
Consensus
SlPI06
SlPI17
SlPI26
SlPI27
SlPI55
SlPI08
SlPI09
SlPI10
SlPI18
SlPI07
Consensus
SlPI02
SlPI11
SlPI12
SlPI13
SlPI14
SlPI15
SlPI16
SlPI52
SlPI54
SlPI51
SlPI53
Consensus
SlPI04
SlPI30
SlPI49
SlPI31
SlPI40
SlPI42
SlPI43
SlPI45
SlPI41
SlPI33
SlPI34
SlPI35
SlPI36
SlPI38
SlPI37
SlPI39
SlPI44
SlPI47
SlPI48
SlPI32
SlPI17
SlPI18
Consensus
